# P-1411. Perinatal Care-Seeking Among Women Living with HIV in Uganda

**DOI:** 10.1093/ofid/ofae631.1586

**Published:** 2025-01-29

**Authors:** Cora Alperin, Esther Atukunda, Cynthia Harper, Charles Baguma, Annet Kembabazi, Alison El Ayadi, Rumbidzai Mushavi, Emily Satinsky, Mercy Juliet, E Betty Namara, Phionah Ahereza, Alexander Tsai, Alison Comfort

**Affiliations:** Baylor College of Medicine, Houston, Texas; Mbarara University of Science and Technology, Mbarara, Mbarara, Uganda; University of California, San Francisco, San Francisco, California; Mbarara University of Science and Technology, Mbarara, Mbarara, Uganda; Mbarara University of Science and Technology, Mbarara, Mbarara, Uganda; University of California, San Francisco, San Francisco, California; Massachusetts General Hospital, Boston, Massachusetts; University of Southern California, Los Angeles, California; Mbarara University of Science and Technology, Mbarara, Mbarara, Uganda; Mbarara University of Science and Technology, Mbarara, Mbarara, Uganda; Mbarara University of Science and Technology, Mbarara, Mbarara, Uganda; Massachusetts General Hospital, Boston, Massachusetts; University of California, San Francisco, San Francisco, California

## Abstract

**Background:**

Inadequate perinatal care uptake in Uganda, as outlined by national guidelines (eight antenatal visits starting before 26 weeks, supervised facility delivery, and at least one postnatal checkup), persists as a significant issue. Factors such as HIV-related stigma further hinder healthcare engagement during this period. This study evaluates differences in antenatal and postnatal care-seeking between women living with HIV (WLHIV) and HIV-negative women in Uganda.

**Methods:**

We performed a cross-sectional study of women 18 years and older (n = 417) living in Nyakabare parish, Southwestern Uganda. The study sample was part of a longitudinal complete population cohort followed since 2014. We collected survey data on care-seeking at most recent pregnancy, including self-reported HIV status and HIV testing, timing of perinatal care, and socio-demographics. Outcomes of interest included antenatal care access (ANC), timing of ANC initiation, ANC visits, and postnatal care (PNC) access. The primary explanatory variable was self-reported HIV status. Analyses were conducted using a binomial logistic regression model with covariate adjustment for age, education, wealth, and village.

**Results:**

On average, women initiated ANC in the second trimester when 4 months pregnant, much later than recommended. Further, women attended half the number of recommended ANC visits (4 of 8 visits). Most women (87%) delivered in a healthcare facility and accessed ANC (98%). There was no significant difference in ANC access, time of initiation of ANC, frequency of ANC visits, or location of delivery by HIV status. WLHIV were 2.6 times more likely to have had a postnatal care visit (adjusted odds ratio [aOR] 2.6, 95% confidence interval [CI]1.1-5.5). 68% of WLHIV sought PNC compared to 40% of women who were HIV-negative.

**Conclusion:**

The data reveal a trend of late ANC initiation and incomplete care among all women regardless of serostatus. However, WLHIV were more likely to access PNC compared to HIV-negative women, potentially indicating effective support and follow-up once initiating ANC. Research exploring why WLHIV more consistently access PNC is needed. Targeted interventions to ensure earlier ANC initiation, including for WLHIV, are crucial and should consider differences in healthcare system engagement.

**Disclosures:**

**All Authors**: No reported disclosures

